# Thin-Film Fracture Behavior for Diketopyrrolopyrrole Semiconducting Polymeric Films

**DOI:** 10.1021/acs.chemmater.5c01388

**Published:** 2025-09-15

**Authors:** Song Zhang, Yunfei Wang, Gage T. Mason, Zhiyuan Qian, Simon Rondeau-Gagné, Xiaodan Gu

**Affiliations:** 1 School of Polymer Science and Engineering, Center for Optoelectronic Materials and Device, 5104 The University of Southern Mississippi, Hattiesburg, Mississippi 39406, United States; 2 Department of Chemistry and Biochemistry, 8637 University of Windsor, Windsor, Ontario N9B3P4, Canada

## Abstract

Fracture energy, which quantifies a material’s resistance to the propagation of a pre-existing crack, is a key parameter for ensuring the mechanical reliability of stretchable organic electronic devices. However, most existing methods, such as a four-point bending fracture energy, utilized for measuring the fracture energy of semiconducting polymeric thin films are complicated by substrate effects, making it challenging to isolate the intrinsic behavior of the film from interfacial influences. In this study, we employed a pseudo free-standing pure shear method to systematically investigate the cohesive fracture energy of poly­(diketopyrrolopyrrole-terthiophene) P­(DPP-T)-based thin films to examine the effects of nanoconfinement, side chain length, degree of crystallinity, and strain rates. This method effectively eliminates substrate interference, enabling a direct assessment of the cohesive fracture energy of P­(DPP-T) thin films. We found that thinner films and those with lower molecular weights exhibited significantly reduced fracture energies due to diminished chain entanglements. Additionally, films with shorter side chains displayed notably higher fracture energies, which were attributed to an increase in the degree of crystallinity. Finally, slower strain rates led to higher fracture energies, consistent with an enhanced stress relaxation. These insights offer practical guidelines for designing mechanically robust semiconducting polymers, contributing to the advancement of reliable, durable, flexible, and wearable electronic devices.

## Graphical abstract

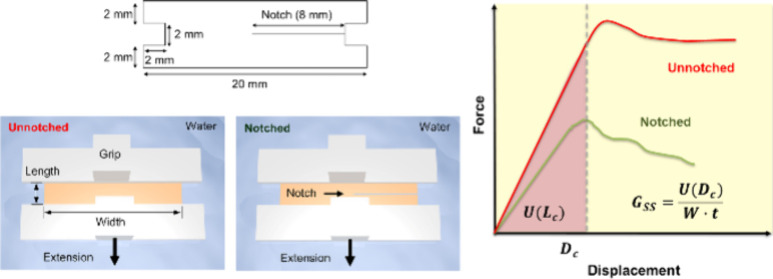

## 1 Introduction

Recently, there has been a key focus in the development of flexible and stretchable organic electronics.^1–10^ While past efforts have primarily focused on enhancing their electronic performance, there is growing interest in engineering these soft, flexible, and mechanically compliant organic electronic devices for conformable technologies compatible with diverse substrates and the human body, enabling new possibilities in wearable electronics. In organic field-effect transistors (OFETs), semiconducting polymers are solution processed into thin films with thicknesses below 100 nm, serving as the active layer that enables charge transport. Thus, a deeper understanding of their mechanical properties and mechanical robustness, on the top of knowledge on their electronic property, would enable the development of new wearable electronic devices.^11–13^

In response to these needs, multiple thin-film mechanical testing methods have been developed and applied to semiconducting polymers, such as the film-on-elastomer buckling method and water-assisted tensile tests.^14–18^ From the stress–strain behavior of polymer thin films, mechanical parameters, such as elastic modulus, yield point, and crack-onset strain, can be readily obtained. A review by Lipomi and co-workers also highlighted the importance of strength, toughness, and elastic range for real-life device applications.^19^ Thus, it is feasible to understand how structural features affect the flexibility, stretchability, elastic reversibility, and hardness of polymeric thin films. However, it remains challenging to quantify the mechanical robustness and reliability with these parameters, whereas crack-onset strain and toughness are extrinsic material properties that can be affected by defect sites (i.e., dust particles and pinhole imperfections) in the film.

As an intrinsic material property, fracture energy measures the ability of a material to resist the propagation of a pre-existing crack.^20–22^ Unlike fracture of brittle materials, which can be described by linear-elastic fracture mechanics (LEFM), most semiconducting polymers are viscoelastic.^23,24^ Those polymers have a strong hysteresis in cyclic stress–strain testing and can exhibit stress relaxation when being pulled.^25^ Thus, both the testing temperature and strain rates can significantly impact and influence the reported modulus and fracture strain. Through the film-on-elastomer buckling method, Lipomi and co-workers quantified the fracture behavior of both brittle and ductile conjugated polymer thin films using scaled crack density for brittle films and microvoid-propagation number for ductile ones.^26^ More direct measurements of thin-film cohesive fracture energy were achieved through four-point bending tests and double-beam cantilever tests, as reported by Dauskardt et al. and further investigated by both Kim et al. and O’Connor et al.^27–32^ In both methods, target thin films were laminated within multiple layers using epoxy, metals, or glass. For the four-point bending test, a notch was first introduced into the glass substrate and extended into the thin films through out of the plane film thickness direction under the applied bending force.^33^ In double-beam cantilever tests, the force was applied on one side of the sample.^34^ The crack initiation point can vary across the film depth and propagates along the in-plane film direction. Thus, the plastic zone is confined to the film surface. For both methods, it was observed that the cohesive fracture energy for the semiconducting polymers ranges from 1 to 10 J/m^2^. However, the effect of the film–substrate interface on the thin-film fracture energy remains unclear.

Recently, our group used pseudo free-standing tensile tests to measure the thin-film fracture energy through the classical Begley–Landes method and the pure shear method.^20^ The high surface energy of water flattens thin films and allows for uniaxial tensile tests. Thus, upon introduction of a notch into the polymeric thin film, the fracture energy can be measured by monitoring the crack propagation and force evolution during the deformation process. Due to the hydrophobicity of test polymers and highly concentrated stress at the notch tip, the potential effects of water diffusion and stabilization are negligible.^35^ Our previous work measured the fracture energy for P3HT and PNDI­(2HD)­T using the Begley–Landes method, where the role of plastic energy dissipation was not fully investigated. The pure shear method, measuring the steady-state fracture energy, can provide more detailed information for the fracture behavior of viscoelastic polymers.^36^

Here, we applied the pure shear method to investigate the thin-film fracture energy of several diketopyrrolopyrrole (DPP)-based semiconducting polymers. Due to their viscoelastic and semicrystalline nature, multiple factors including the chain entanglement (either due to finite film thickness induced confinement effort and molecular weight), different side chain lengths, the degree of crystallinity, and strain rate were investigated to understand influences on the thin-film fracture energy. P­(DPP-T-C_2_C_12_C_14_) was chosen as a model system due to its excellent electronic performance and well-characterized thermomechanical properties through years of collaborations among all coauthors. This is also the first in-depth fracture energy study of conjugated polymeric thin films below 100 nm. These findings shed light on the design principles for mechanically robust semiconducting polymers.

## 2 Experimental Section

### 2.1 Thin-Film Dog-Bone Sample Processing

Conjugated polymer thin films with a thickness of >80 nm were prepared using a spin coating method. The sample solutions, dissolved in chlorobenzene, were cased on the PSS-coated silicon substrate as described in our previous work.^24^ The film thicknesses for the polymers with molecular weights of approximately 40, 60, and 75 kg/mol were 86, 88, and 64 nm, respectively. To achieve comparable film thicknesses, different solution concentrations were used: 20 mg/mL for 40 kg/mol, 15 mg/mL for 60 kg/mol, and 10 mg/mL for 75 kg/mol. All thicknesses are above the critical thickness. Therefore, confinement effects on the mechanical properties should be minimal, as supported by our previous report.^37^

A H-shape dog-bone geometry was then patterned on the sample using a 1064 nm laser. A CAD file was first generate based on the shape shown in Figure 1a, with a gauge section measuring 16 mm in width and 2 mm in length and a pad section measuring 20 mm in width and 2 mm in length. Notched samples were prepared by introducing an 8 mm-long notch along the edge of each sample. The laser etch was next applied. Thermally annealed samples were heated on a hot plate inside a glovebox under a N_2_ atmosphere for 1 h, followed by slow cooling. The annealing temperature used ranged from 100 to 200 °C.

**Figure 1:**
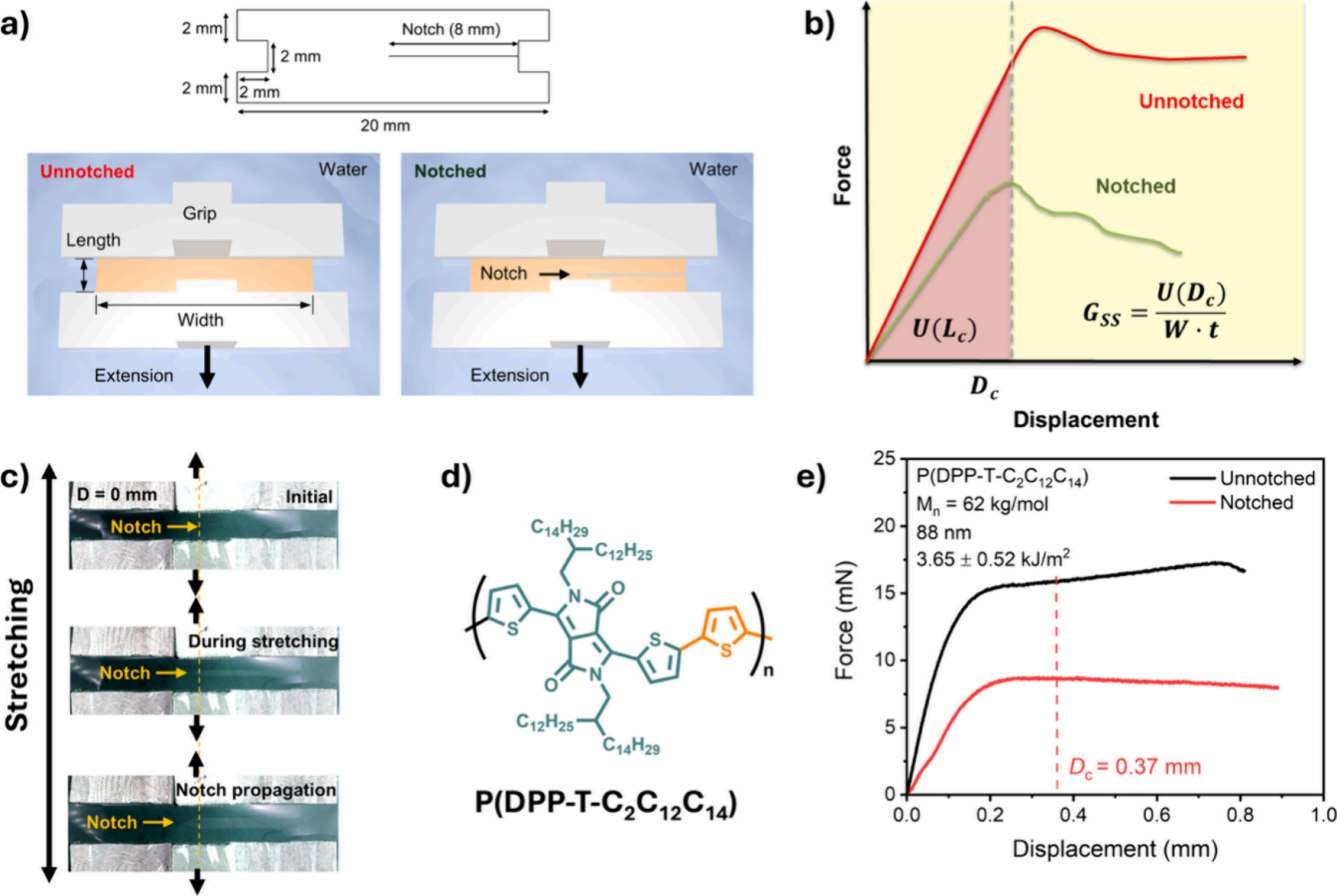
(a) Schematic of the fracture energy test setup with a rectangular notched polymeric thin sample attached to grips in the film width direction. The dimension of the test sample is shown. (b) Representative force–displacement curves for unnotched and notched samples and the calculation of fracture energy (*G*_ss_), where *U*(*D*_c_) is the area under the stress–strain curve for the unnotched sample and *W* and *t* are the width and film thickness for the thin film sample, respectively. (c) Representative optical images of notch growth during uniaxially deformation at different displacements. (d) Chemical structure of P­(DPP-T-C_2_C_12_C_14_) polymer. (e) Force–displacement curves for unnotched and notched P­(DPP-T-C_2_C_12_C_14_) films with a number-averaged molecular weight (*M*_n_) of 62.8 kg/mol. *D*_c_ represents the critical displacement. The area within the red box is zoomed in to show the maximum force point.

### 2.2 Fracture Energy Tests

Pseudo free-standing tensile tester was utilized to measure the stress–strain behavior of both unnotched and notched samples, following a previously reported method by our group.^20^ A strain rate of 0.01 mm/s was applied, unless otherwise specified. The critical distance *D*_c_ for area integration was defined as the point at which the maximum force was reached in the stress–strain curve of notched samples.

### 2.3 Grazing Incidence Wide-Angle X-ray Scattering (GIWAXS) Measurements

GIWAXS measurements of polymeric thin films on a the silicon substrate were performed using a laboratory beamline system (Xenocs Inc. Xeuss 2.0*)* with an X-ray wavelength of 1.54 Å and a sample-to-detector distance of 150 mm. An incidence angle of 0.2° was used. Samples were kept under a vacuum to minimize air scattering. Diffraction images were recorded on a Pilatus 1 M detector (Dectris Inc.) with an exposure time of 1 h and processed using the Nika software package in combination with WAXSTools. To calculate the relative degree of crystallinity (rDOC), the intensity of the (100) peak was normalized by exposure time, sample thickness, and beam path length. Then, geometrically corrected orientation distribution function, sin­(χ)­I­(χ), was calculated to determine the relative orientation of the crystallite. rDOC was obtained by integrating the area below each scattering profile.

### 2.4 Solution Viscosity Measurements

Semiconducting polymers were dissolved in chlorobenzene at a concentration of 10 mg/mL and stirred overnight at 80 °C prior to measurements. Solution viscosity measurements were conducted by using an AR1500ex rheometer (TA Instruments, Inc.) equipped with a Peltier temperature control system. A cone-and-plate geometry with a 40 mm diameter and a 1° cone angle were used. Measurements were carried out at 25 °C at a shear rate of 100 s^–1^ and a sampling time of 15 s.^38^ Viscosity was first performed in deionized water and chlorobenzene, and the value showed great agreement with literature reports, within 3–5% deviation.^39,38^ Subsequently, the viscosity of the polymer solutions was measured using the same protocol. The specific viscosity (η_sp_) of polymer solution was calculated using the following relationship: $\eta_{\mathrm{sp}}=\frac{\eta -\eta_{s}}{\eta_{s}}$ , where η and η_s_ are the viscosity of polymer solution and pure chlorobenzene, respectively.^40^

## 3 Results

### 3.1 Steady-State Fracture Energy Test

Steady-state fracture energy (also known as the critical energy release rate) is a material property that quantifies the energy required to propagate a crack per unit area under mode I (tensile opening) fracture.^41–44^ In the pure shear test, it is measured under a specific geometry and loading mode designed to create a uniform and constant stress field near the crack tip, allowing for stable crack propagation. In the pure shear test, a notched specimen is used to initiate and control crack propagation, and an unnotched specimen is used to measure the stored strain energy per unit volume. This helps isolate the fracture energy purely associated with crack growth and not with stress concentrations or artifacts of the notch.

The semiconducting polymer thin film was prepared by spin coating polymer solutions in chlorobenzene (CB) between 5 and 15 mg/mL, followed by laser ablation to form a dog-bone-shaped sample geometry. As shown in Figure 1a, an H-shaped dog-bone thin film was floated on the water surface, with a pad size of 20 mm (width, *W*) by 2 mm (length, *L*) and a gauge size of 16 mm (width) by 2 mm (length). Importantly, a width-to-length ratio of 8 was used to ensure the validity of the pure shear geometry. In addition, a side-notched sample was prepared by introducing an 8 mm-long notch parallel to the width direction (Figure 1a). After the thin film was transferred onto the water surface and attached to the grips of the tensile stage, a uniaxial tensile test was performed. Two types of force–displacement curves were obtained for the notched and unnotched samples, respectively (Figure 1b). The stress–strain curve of the notched sample is presented to show the selectivity of critical displacement (*D*_c_), the point of maximum force where the notch transitions into a propagating crack. From the force–displacement curve of the notched sample, a *D*_c_ was identified at the point of maximum force, where the notch transitions into a propagating crack. Steady-state fracture energy (*G*_ss_) was calculated based on the stress–strain curve of the unnotched sample from the following equation:

$$G_{\mathrm{ss}}=\frac{U(D_{c})}{W\cdot t}$$

where *U*(*D*_c_) is the area under the stress–strain curve for the unnotched sample, *W* is the sample width, and *t* is the film thickness.^20^ A representative image of the growing notch during stretching is shown in Figure 1c.

P­(DPP-T-C_2_C_12_C_14_) with a number-average molecular weight (*M*_n_) of 61.8 kg/mol was used as a model polymer for our investigation (Figure 1d). Figure 1e shows the corresponding force–displacement curve at a stretching rate of 0.01 mm/s. A film thickness above 80 nm was selected to avoid a strong thin film confinement effect. For the unnotched sample, a typical viscoelastic response was observed. The force increased continuously until reaching a crack onset strain of approximately 40%, or 0.8 mm displacement. For the notched sample, after the initial elastic region, the maximum force occurred at a *D*_c_ of 0.37 mm, followed by a graduate decrease. Consequently, a *G*_ss_ value of 3.65 kJ/m^2^ was obtained. This value is comparable to that of glassy polystyrene, which exhibits a *G*_ss_ of 4 kJ/m^2^ from finite element simulation.^20^

### 3.2 Nanoconfinement Effect

Nanoconfinement is recognized as a critical factor influencing the mechanical properties of polymeric films, particularly with respect to the film thickness and polymer chain length. In our recent work, we discussed how elastic modulus and crack onset strain can be influenced by thin film confinement.^37^ The increased mobility of polymer chains at the air–film interface compared to the bulk phase, combined with reduced interchain entanglements and enhanced intrachain interactions when the film thickness is below the polymer chains’ end-to-end distance (*R*_e__e_), underscores the importance of thickness as an important tuning parameter for controlling the degree of nanoconfinement. For example, studies have reported that reduced film thickness in conjugated polymers such as P3HT and P­(DPP-T) leads to a significant decrease in stretchability due to a loss of entanglements.^37^ Similarly, in traditional polymers such as polystyrene, fracture energy decreases as film thickness approaches *R*_e__e_, a phenomenon attributed to diminished interchain entanglement under nanoscale confinement.^20,45–47^ These findings suggest that thickness-dependent entanglement behavior likely influences the fracture behavior of semiconducting polymers also. P­(DPP-T-C_2_C_12_C_14_) thin films with various thickness ranging from 35 to 96 nm were prepared by adjusting the solution concentration and spin-coat rates (Figure 2a and Figure S1). As the film thickness increased from 35 to 64 nm, the fracture energy rose from 1.93 to 5.56 kJ/m^2^ with increased *D*_c_ from 0.26 to 0.63 mm, which represents an enhancement of approximately 3-fold. For thicker films from 64 to 96 nm, the fracture energy slightly increases to 6.59 kJ/m^2^ with increased *D*_c_ to 0.83 mm. Our previous study showed that the critical thickness for confinement of P­(DPP-T) with a similar molecular weight is approximately 40 nm.^37^ Therefore, the reduction in fracture energy for the thinnest film and *D*_c_ is attributed to a loss of chain entanglement.

**Figure 2:**
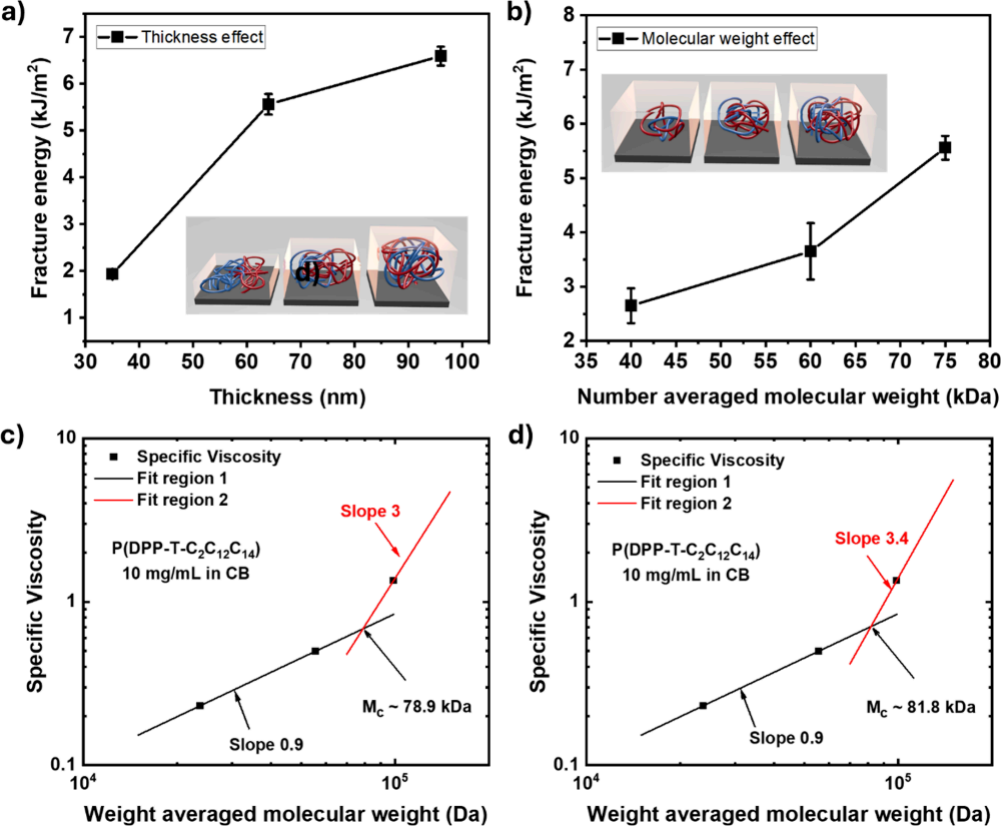
Nanoconfinement effect on the fracture energy of P­(DPP-T-C_2_C_12_C_14_). (a) Fracture energy of P­(DPP-T-C_2_C_12_C_14_) with different thicknesses of 35, 64, and 96 nm. (b) Fracture energy of P­(DPP-T-C_2_C_12_C_14_) with different molecular weights. Critical entanglement molecular weight (*M*_c_) of P­(DPP-T-C_2_C_12_C_14_) determination by solution rheology using power laws of (c) 3 and (d) 3.4. The weight-averaged molecular weight (*M*_w_) is used for the data analysis here for parts c and d for the solution viscosity study.

In addition to film thickness, the molecular weight also plays an important role in fracture energy. As molecular weight increases, the number of entanglements per chain also increases, leading to higher elastic moduli, crack-onset strain, toughness, and charge carrier mobility, until a saturation regime is reached.^19,48–51^ Thus, P­(DPP-T-C_2_C_12_C_14_) thin films with average weight molecular weight *M*_w_ values of 48–109 kDa were synthesized to investigate the effect of molecular weight on fracture energy. However, the P­(DPP-T-C_2_C_12_C_14_) thin films with low *M*_w_ values were found to be too brittle to be measured. Therefore, only the ones with *M*_w_ higher than 95 kDa were measured, which increased with increasing MW, reaching 2.65, 3.65, and 5.56 kJ/m^2^ for the P­(DPP-T-C_2_C_12_C_14_) film with *M*_*n*_ of 40, 60, and 75 kDa, respectively (Figure 2b and Figure S2).

To investigate the influence of the above observed molecular weight on fracture energy, polymer solution rheology measurements were conducted to assess the impact of chain length on the entanglement behavior of P­(DPP-T-C_2_C_12_C_14_).^52^ According to reptation solution theory,

$$\eta -\eta_{s}\approx G_{e}\tau_{\mathrm{rep}}\approx \eta_{s}\frac{N^{3}}{[N_{e}(1){]}^{2}}\{\begin{array}{c} \varphi^{3/(3v-1)}\;\mathrm{for}\;\mathrm{an}\;\mathrm{athermal}\;\mathrm{solvent}\\ \varphi^{14/3}\;\mathrm{for}\;a\;\theta -\mathrm{solvent} \end{array}$$

where η and η_s_ are the viscosities of solution and solvent, respectively, ϕ is the concentration of polymer solution, *N* is the degree of polymerization (DP), and *N*_e_ is the entanglement DP.^54^ Besides, the specific viscosity is calculated by the following equation:

$$\eta_{\mathrm{sp}}=\frac{\eta -\eta_{s}}{\eta_{s}}$$

Therefore, when the same polymer, solvent, and polymer solution concentrations are used, η_sp_ *∼ N* ^3^. However, experimental results often report a higher exponent of ∼3.4 due to additional dynamic effects. Here, the specific viscosity (η_sp_) for a polymer solution with a different *M*_w_ was first measured using a rheometer.^40,41^ Figure 2c shows the dependence of η_sp_ on the weight-average molecular weight (*M*_w_) for P­(DPP-T-C_2_C_12_C_14_) dissolved in chlorobenzene at a concentration of 10 mg/mL. A power-law exponent of 0.9 was obtained in the low *M*_w_ region (region 1). In the high *M*_w_ region (region 2), a power law of 3, indicative of reptation scaling as expected, was used to qualitatively investigate the entanglement molecular weight.^53^ Hence, the critical weight-averaged molecular weight (*M*_c_) of 78.9 kg/mol was calculated from the intersection of those two lines. For regular flexible polymers, a power law of 3.4, stronger than reptation scaling, is generally observed in the high *M*_*w*_ region. Therefore, the extrapolation of measured data point in the high *M*_*w*_ with a known power law of 3.4 was also applied, as shown in Figure 2d. Thus, from the intersection, a critical weight-average *M*_w_ of 81.8 kg/mol was observed. Unlike other nonconjugated polymers, where the controlled living polymerization was developed, conjugated polymers are typically synthesized through polycondensation using cross-coupling reactions such as Stille polycondensation and Suzuki–Miyaura coupling, which generally results in poor control of the molecular weight and high dispersity.^54–56^ Since all measured weight-averaged molecular weights are above *M*_c_, the fracture energy increases continuously with *M*_w_. Since all measured weight-averaged molecular weights are above *M*_c_, the fracture energy increases continuously with *M*_w_, without a sharp transition from brittle to ductile here in Figure 2b.

To verify the observed molecular effect, the *M*_w_ dependence of η_sp_ for another synthesized DPP-based polymer, namely, P­(DPP-TVT-C_2_C_12_C_14_) (10 mg/mL in chlorobenzene), was measured here. The finding is plotted in Figures S3. A power law of 1.4 was observed for η_sp_ at the low *M*_w_ region. In the high *M*_w_ region, again, the data extrapolation with the *M*_w_ data point was applied. With a power of 3, a *M*_c_ of 87.5 kg/mol was calculated from the intersection of those two lines. Alternatively, the *M*_c_ of 91 kg/mol can be obtained if one assumes a power law of 3.4 for viscosity and molecular weight dependence. This is approximately 10 kg/mol higher than the *M*_c_ of P­(DPP-T-C_2_C_12_C_14_), which is likely due to their different backbone rigidity. Future solution neutron scattering is needed to confirm this hypothesis, since the focus of this work is on the thin film fracture behavior.

### 3.3 Side-Chain Length Effect

For semiconducting polymers, the side chain length greatly affects the thermal and mechanical response. Here, we also systematically studied the impact of side chain length on fracture energy by investigating four DPP-T-based semiconducting polymers with the same backbones but different side chains. These four polymers were used in our previous publication to study their glass transition temperature (*T*_g_) and elastic modulus.^57^ We observed a decrease in backbone *T*_g_ and elastic modulus with increasing side chain length from C_2_C_6_C_8_ (2-hexyl decyl) to C_2_C_12_C_14_ (2-dodecyl hexadecyl), while the crack onset strain did not show a clear dependence.^57^ Here, fracture energy tests were performed on four DPP polymers, P­(DPP-T-C_2_C_6_C_8_), P­(DPP-T-C_2_C_8_C_10_), P­(DPP-T-C_2_C_10_C_12_), and P­(DPP-T-C_2_C_12_C_14_), all having similar weight-averaged molecular weights of approximately 60–80 kg/mol (Figure 3 and Figure S4). For unnotched samples, the strain-hardening region at displacements above 0.5 mm showed a shallower slope with increasing side chain length, due to reduced entanglement density, as reported previously.^58^ Under a strain rate of 0.01 mm/s, the critical crack propagation length *D*_c_ increased from 0.67 mm P­(DPP-T-C_2_C_6_C_8_) to 1.15 mm P­(DPP-T-C_2_C_8_C_10_) and then 1.26 mm P­(DPP-T-C_2_C_10_C_12_). For P­(DPP-T-C_2_C_6_C_8_) and P­(DPP-T-C_2_C_10_C_12_), the crack onset strain for unnotched samples was slightly lower than that for the corresponding *D*_c_, so an auxiliary line was added to extend the force–displacement curve. In contrast to *D*_c_, the fracture energy decreased from 32.10 kJ/m^2^ (P­(DPP-T-C_2_C_6_C_8_) to 29.31 kJ/m^2^ P­(DPP-T-C_2_C_8_C_10_) and then to 27.19 kJ/m^2^ P­(DPP-T-C_2_C_10_C_12_). In addition to differences in entanglement density, such variation may also result from morphological differences, such as the degree of crystallinity and difference chain mobilities, reflected by different testing temperatures *T* and sample *T*_g_. A detailed study on the influence of DOC on fracture energy is presented in the next section. Interestingly, the fracture energy of these three polymers is 1 order of magnitude higher than that of P­(DPP-T-C_2_C_12_C_14_), which could result from its much lower elastic modulus, leading to a much lower *D*_c_.

**Figure 3:**
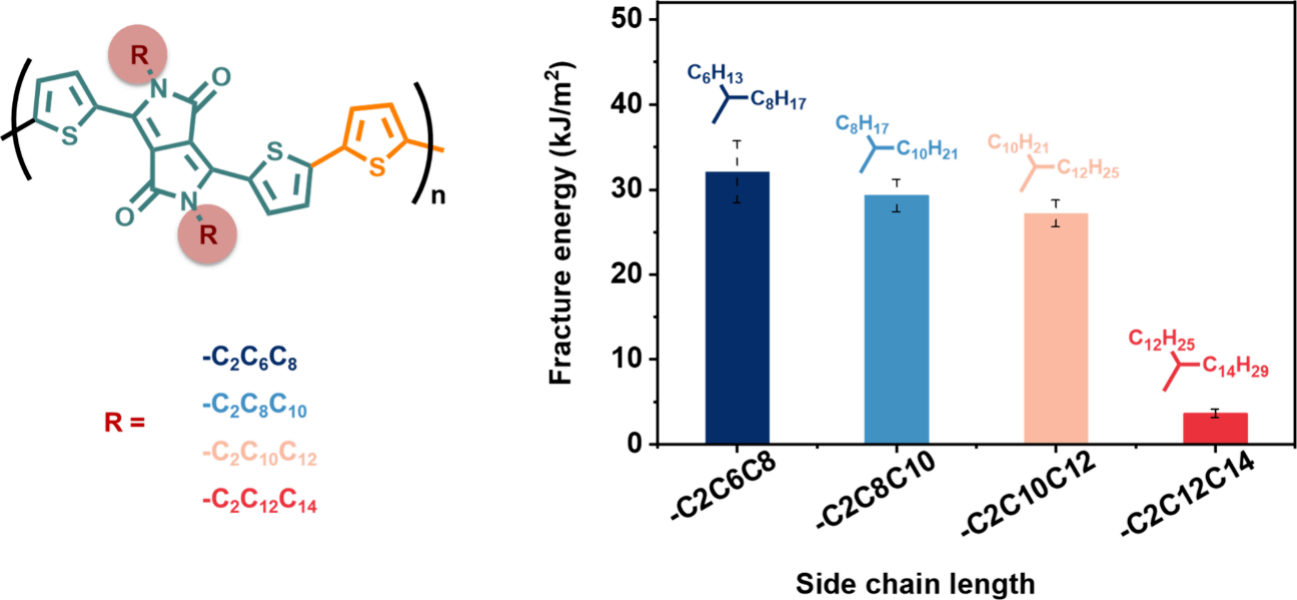
Side chain length effect on the fracture energy of P­(DPP-T-C_2_C_12_C_14_). Chemical structure and fracture energy of PDPPT conjugated polymers with side chains −C_2_C_6_C_8_, −C_2_C_8_C_10_, −C_2_C_10_C_12_, and −C_2_C_12_C_14_.

### 3.4 Degree of Crystallinity Effect

Next, we investigated the effect of crystallinity on the fracture energy. To study this effect, P­(DPP-T-C_2_C_12_C_14_) thin films were thermally annealed at 100 °C, 150 °C, and 200 °C to promote their crystallization after spin-casting (Figure 4). The relative degree of crystallinity was quantified using grazing incidence wide-angle X-ray scattering (GIWAXS, Figure S5), which revealed increased crystallinity with higher annealing temperatures. The sample annealed at 200 °C showed approximately 2.6 times greater crystalline content. Fracture energy measurements of the annealed thin films showed a direct correlation with the degree of crystallinity (DOC), as higher degrees of crystallinity resulted in increased fracture energy (from 5.6 to 9 kJ/m^2^, Figure 4 and Figure S6). From our previous work, the backbone *T*_g_ of PDPP-T-C2C12C14 is −13.26 °C measured by the substrate-supported DMA method.^57^ More recent work suggests that the backbone twisting of the DPP polymer happens around 150 °C or high.^59^ Above this temperature, PDPP-T-C2C12C14 exhibits more backbone motion. At higher annealing temperatures (e.g., 200 °C), increased polymer chain mobility facilitates more ordered packing, enhancing the degree of crystallinity (DOC) and resulting in the higher degree of crystallinity and stiffness of resulting films.^58^

**Figure 4:**
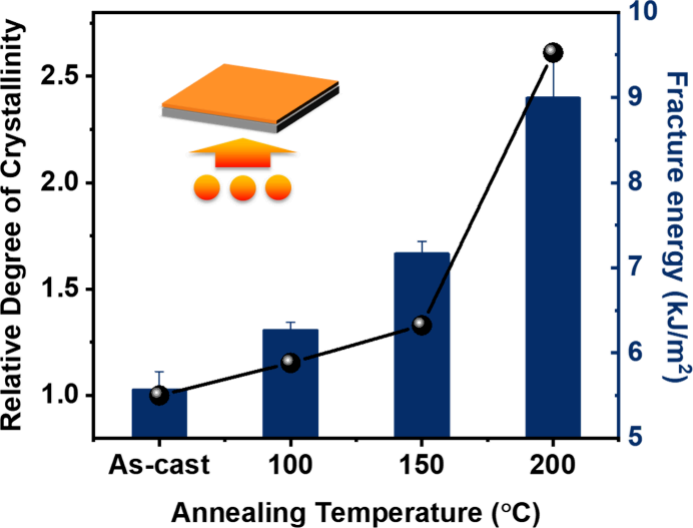
DOC effect on the fracture energy of P­(DPP-T-C_2_C_12_C_14_). Summarization of the relative degree of crystallinity (rDOC) and fracture energy for the as-cast and thermally annealed P­(DPP-T-C_2_C_12_C_14_) thin films at 100, 150, and 200 °C.

### 3.5 Strain Rate Effect

Finally, the strain rate effect was investigated, as it has been previously reported to influence mechanical response.^24^ Different strain rates were studied including 0.0025, 0.005, 0.025, and 0.05 s^–1^ (Figure 5 and Figure S7). At the slowest strain rate, the P­(DPP-T-C_2_C_12_C_14_) thin film showed the highest fracture energy, which was attributed to the viscoelastic nature of the material. The sample simply had more time to rearrange its chain segments and allowed greater energy dissipation during tensile deformation. With increasing strain rate, a trade-off between the increased elastic modulus and reduced fracture strain was observed. This trade-off canceled off the change in elastic modulus and fracture strain and resulted in a weak dependence between the strain rates and fracture energy between 0.005 and 0.05 s^–1^. Overall, the fracture energy showed a weak dependence on the strain rates, mainly due to the viscoelastic nature of the DPP polymers.

**Figure 5:**
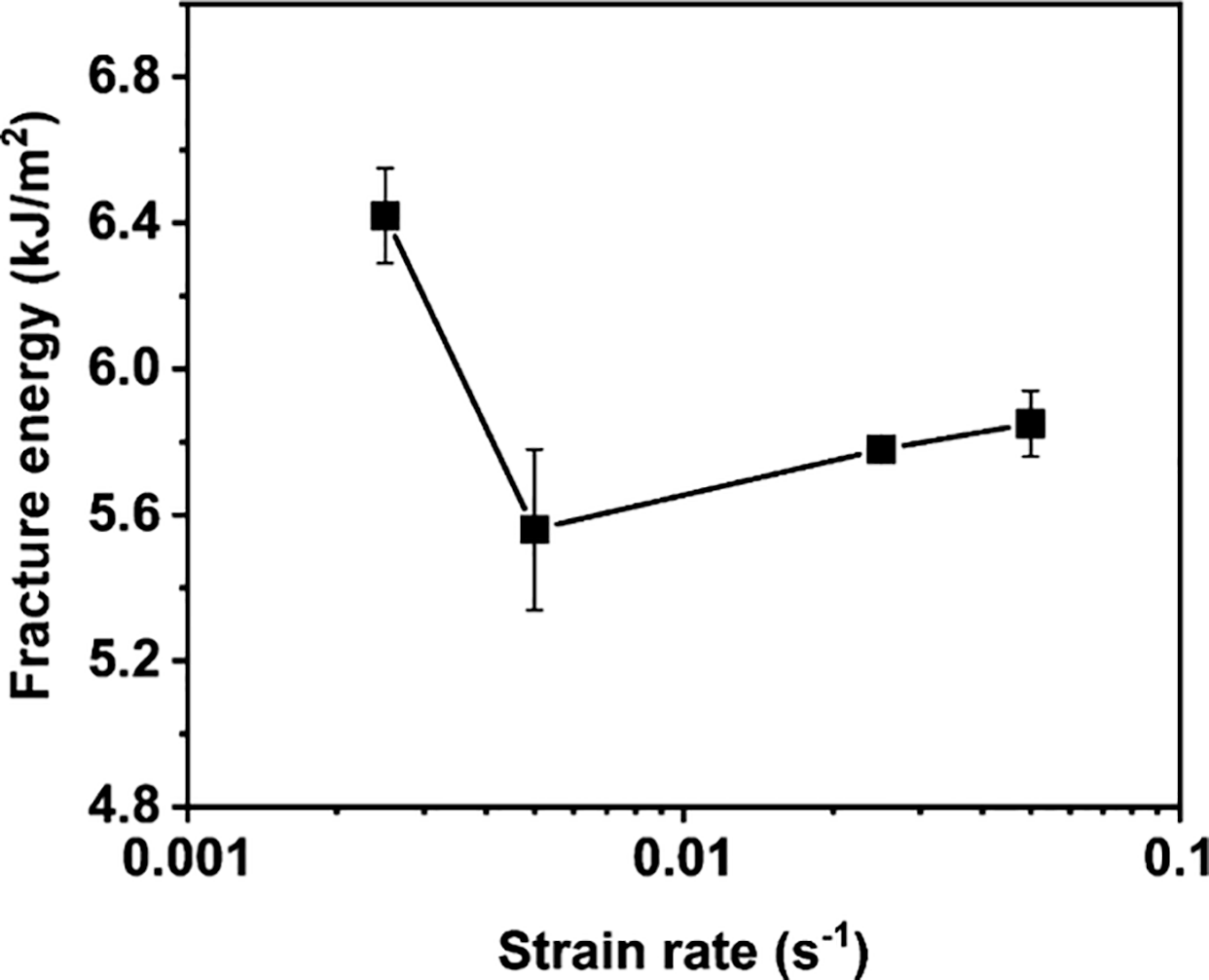
Fracture energy of P­(DPP-T-C_2_C_12_C_14_) at strain rates of 0.0025, 0.005, 0.025, and 0.05 s^–1^.

## 4 Discussion

In this work, we offered the fracture energy as an alternative metric to analyze the mechanical properties of conjugated polymers, allowing us to effectively eliminate substrate interference and enabling the precise measurement of the sample’s ability to resist crack propagation. This method is different from the commonly used crack on strain (COS) method to gauge the deformability of the polymer thin film. There are pros and cons when comparing those two metrics.

COS and fracture energy are both critical metrics for assessing mechanical failure in conjugated polymer films under tension, but they capture different aspects of deformability. COS is defined as the tensile strain at which the film first develops observable cracks, essentially serving as a measure of film ductility or stretchability. It is typically determined by stretching a thin polymer film (often on a flexible substrate) until microcracks appear using optical or AFM imaging to detect the onset of cracking. This threshold indicates how much strain the film can accommodate before its continuous integrity is compromised. It is very straightforward to understand and relatively easy to study. Fracture energy, *G*_c_, outlined in this work, however, is a material’s cohesive toughness—the energy per unit area required to propagate a crack through the film. In contrast to the fracture strain, it has a unit of J/m^2^ and is obtained from fracture mechanics tests (such as four-point bending, double-cantilever beam setups, or the pure shear method used in this work) that measure how much energy the film absorbs before complete failure. In essence, COS provides a strain limit for crack initiation, whereas fracture energy provides a quantitative gauge of resistance to crack propagation. Both parameters are related: a polymer film that can sustain a higher COS without cracking often also exhibits a higher fracture energy, since delaying the crack onset usually correlates with greater energy absorption capacity. However, they are not interchangeable. COS is a one-point threshold (the onset of damage), while fracture energy reflects the integrative toughness of the material beyond that point. A film could, for example, have a moderately low crack onset strain yet still possesses a relatively high fracture energy if it undergoes substantial plastic deformation or crack blunting after the initial microcracks form. This is the example for our P­(DPP-T) with the shortest side chains and high degrees of crystallinity. Conversely, a very high COS material that fails in a brittle manner once that threshold is passed would have limited fracture energy. Thus, COS and fracture energy provide complementary views of mechanical robustness: the former emphasizes when cracks start, and the latter quantifies how much energy is needed to drive those cracks to failure.

When evaluating the deformability of semiconducting polymer films for flexible electronics, here, we propose that each parameter offers distinct advantages and limitations. COS is a straightforward method to measure and is widely used as a quick indicator of stretchability in thin-film devices. Its simplicity (stretch-and-observe) makes it an effective screening tool during materials development; researchers often report COS to compare how different polymer chemistries or processing methods improve film ductility. For instance, brittle inorganic layers like silicon or gold metal electrode have COS on the order of few percents of strain (e.g., <5%), whereas conjugated polymer films can sustain strains of tens percent to over 100% before cracking. Such a dramatic differences underlines why high COS is prized for wearable and stretchable electronics: a device can endure much larger deformations before any cracks initiate that would disrupt electrical continuity. The main limitation of COS is that it provides a relatively qualitative measure of deformability. The exact COS value can depend on how cracks are detected and defined (microscope resolution, focus of the image, crack size threshold, etc.), and thus, comparing COS across different studies requires caution. The substrate interaction with the film, as well as the encapsulation layer, also has an impact on the measurement. Moreover, COS captures the onset of damage but not the postonset behavior; it says little about how the material behaves after the initial cracks form. Fracture energy *G*_c_, in contrast, offers a quantitative and more comprehensive measure of mechanical toughness, which is particularly important for understanding the long-term reliability. A high *G*_c_ means that the film can absorb more mechanical energy (through mechanisms such as chain elongation, entanglement slippage, or crazing) before a crack can propagate catastrophically, indicating robust mechanical integrity. In practice, there are likely defects within the sample that cause a hot spot for stress accumulation. In this sense, if the energy during the tensile stretch is lower than the *G*_c_, the defect is stable and will not propagate. This makes fracture energy a crucial metric for flexible electronics that undergo repeated bending or stretching, as it correlates with the material’s ability to resist failure under stress beyond just initial cracks. The drawback is that measuring *G*_c_ is more complex and time-consuming; it requires specialized mechanical tests and often meticulous sample preparation (to create and propagate a precrack in a controlled way). Additionally, *G*_c_ was used to quantify fracture toughness, and we also note that in polymeric systems, it often encapsulates dissipative and rate-dependent effects beyond linear elasticity. Recent works on nonlinear fracture mechanics in thin films were reported by the Kim group to further the fracture analysis of thin films.^33,34^ As a result, COS is far more common in routine characterization, while *G*_c_ is determined in more in-depth studies, as this work suggests. Despite the practical challenges, we argue that both parameters in mechanical characterization provide a fuller picture: COS tells us the strain limit to avoid crack initiation in operation, and fracture energy tells us how forgiving the material is to flaws or cracks that do occur. In the context of soft and stretchable electronics, balancing these metrics is key. An ideal semiconducting polymer film for flexible devices would combine a very high COS (to prevent any cracks under normal use) with a high *G*_c_ (to ensure that if cracks do form, they grow slowly and require significant energy, thereby preventing abrupt failure). By considering both the COS and *G*_c_, researchers can design and evaluate conjugated polymers that not only stretch to high strains without damage but also resist fracture propagation, offering both flexibility and durability for next-generation flexible electronic applications.

## 5 Conclusions

In summary, we employed a pseudo free-standing pure shear method to systematically evaluate the cohesive fracture energy of P­(DPP-T) based thin films, focusing on the effects of nanoconfinement, side-chain length, crystallinity, and strain rate. Our results reveal that films approaching the critical thickness and those made from lower-molecular-weight polymers exhibit markedly reduced fracture energy, largely due to diminished chain entanglement under nanoscale confinement. This finding is in line with previously observed findings in the community that thinner films become increasingly brittle. Conversely, polymers with shorter side chains demonstrate significantly higher fracture energy, which are attribute to their increased crystallinity and higher elastic modulus. Additionally, the slowest strain rates enhance fracture energy, consistent with improved stress relaxation over time. These findings provide valuable design principles for developing mechanically robust semiconducting polymers, supporting the advancement of reliable, durable, flexible, and wearable electronic devices.

## Supplementary Material



## Notes

Additional experimental details: Force–displacement curves for unnotched and notched P­(DPP-T-C2C12C14) films with different thicknesses, molecular weights, at different strain rates and after annealing at different temperatures; Critical molecular weight (*M*_c_) of P­(DPP-TVT-C2C12C14) determined by the solution rheology measurement; Force–displacement curves for unnotched and notched P­(DPP-T) polymers with different side chains (a) −C_2_C_6_C_8_, (b) −C_2_C_8_C_10_, and (c) −C_2_C_10_C_12_ thin films; GIWAXS results for the as-cast and thermally annealed P­(DPP-T-C_2_C_12_C_14_) thin films (PDF)
