# Supplementary material for: Thin-Film Fracture Behavior for Diketopyrrolopyrrole Semiconducting Polymeric Films
Source: Chem Mater. 2025 Sep 15;37(19):7804–12. doi: 10.1021/acs.chemmater.5c01388 (PMC12529905; doi:10.1021/acs.chemmater.5c01388)
Supplement: Supplementary file 1 [file cm5c01388_si_001.pdf]

Supporting Information

Thin-film fracture behavior for diketopyrrolopyrrole semiconducting polymeric films

*Song Zhang,[1#] Yunfei Wang,[1#] Gage T. Mason[2], Zhiyuan Qian[1], Simon Rondeau-Gagné[2], Xiaodan Gu[1]**

[1]School of Polymer Science and Engineering, Center for Optoelectronic Materials and Device, The University of Southern Mississippi, Hattiesburg, MS 39406, USA

E-mail: xiaodan.gu@usm.edu

[2]Department of Chemistry and Biochemistry, University of Windsor, Windsor, Ontario, N9B3P4, Canada

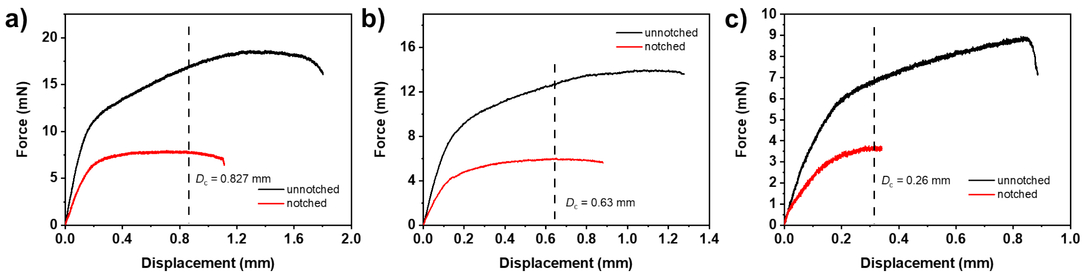

Thickness = 96 nm
G_{ss} = 6.59 ± 0.20 $KJ\,m^{-2}$

Thickness = 64 nm
G_{ss} = 5.56 ± 0.22 $KJ\,m^{-2}$

Thickness = 35 nm
G_{ss} = 1.93 ± 0.03 $KJ\,m^{-2}$

Figure S1. Force−displacement curves for unnotched and notched P(DPP-T-$C_2C_{12}C_{14}$) films with the thickness of a) 96 nm, b) 64 nm and c) 35 nm. D_c represents the critical displacement. Stretching rate used here is 0.01 mm/s.

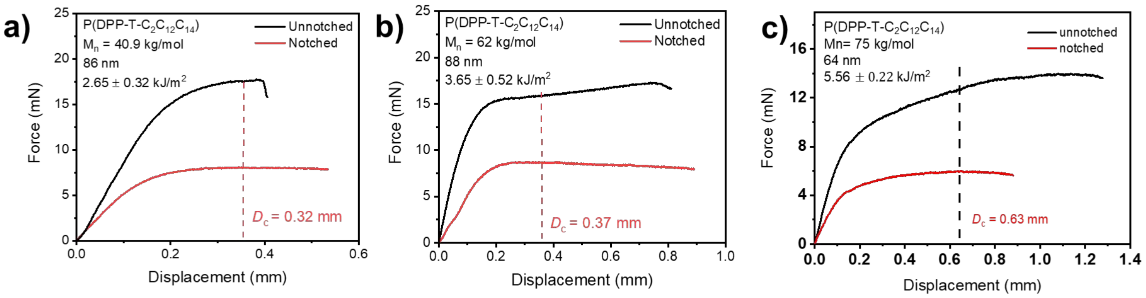

MW = ~40 kDa
G_{ss} = 2.65 ± 0.32 $KJ\,m^{-2}$

MW = ~60 kDa
G_{ss} = 3.65 ± 0.52 $KJ\,m^{-2}$

MW = ~75 kDa
G_{ss} = 5.56 ± 0.22 $KJ\,m^{-2}$

Figure S2. Force−displacement curves for unnotched and notched P(DPP-T-$C_2C_{12}C_{14}$) films with the molecular weight of a) ~40 kDa, b) 60 ~kDa and c) ~75 kDa. D_c represents the critical displacement. Stretching rate used here is 0.01 mm/s.

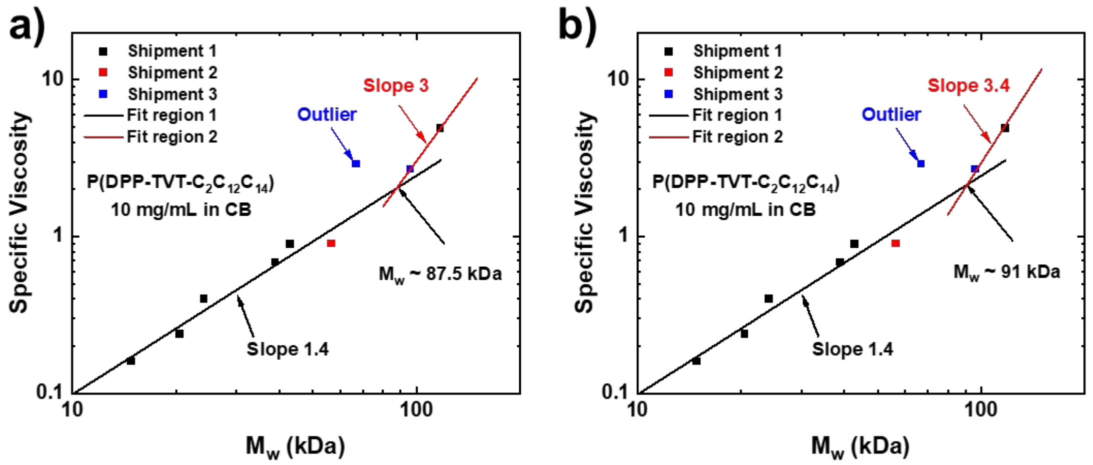

Figure S3. Critical molecular weight (M_c) of P(DPP-TVT-$C_2C_{12}C_{14}$) determined by the solution rheology measurement using the power law of a) 3 and b) 3.4.

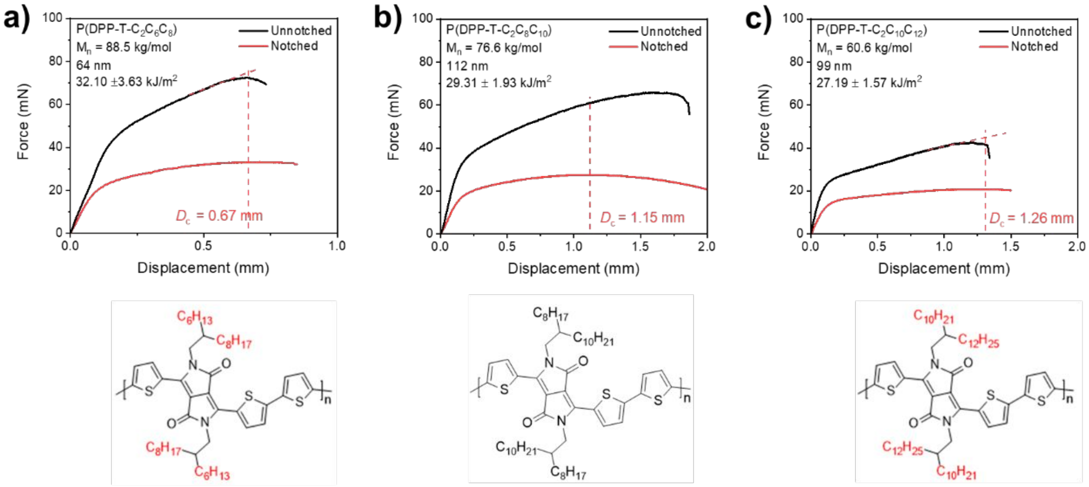

$G_{ss} = 32.10 \pm 3.63\ KJ\ m^{-2}$ $G_{ss} = 29.31 \pm 1.93\ KJ\ m^{-2}$ $G_{ss} = 27.19 \pm 1.57\ KJ\ m^{-2}$

Figure S4. Force−displacement curves for unnotched and notched P(DPP-T) polymers with different sidechains (a) -$C_2C_6C_8$, (b) -$C_2C_8C_{10}$ and (c) -$C_2C_{10}C_{12}$ thin films. D_c represents the critical displacement. Stretching rate = 0.01 mm/s. The molecular weight for P(DPP-T) polymers with side chain of -$C_2C_6C_8$, -$C_2C_8C_{10}$ and -$C_2C_{10}C_{12}$ -$C_2C_{12}C_{14}$ are 88.5, 76.6, 60.6 and 61.8 kDa.

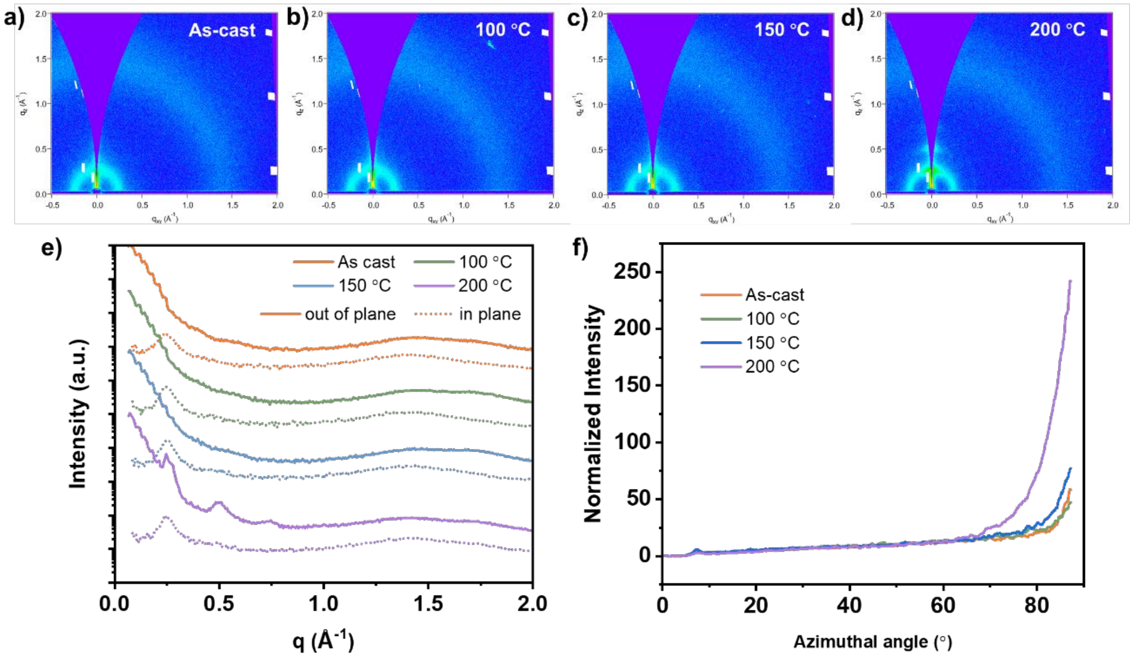

Figure S5. GIWAXS for as cast and thermal annealed P(DPP-T-$C_2C_{12}C_{14}$) thin films. (a-d) 2D images, (e) 1D profile and (f) pole figure for as cast and thermal annealed P(DPP-T-$C_2C_{12}C_{14}$)thin films at 100 °C, 150 °C and 200 °C.

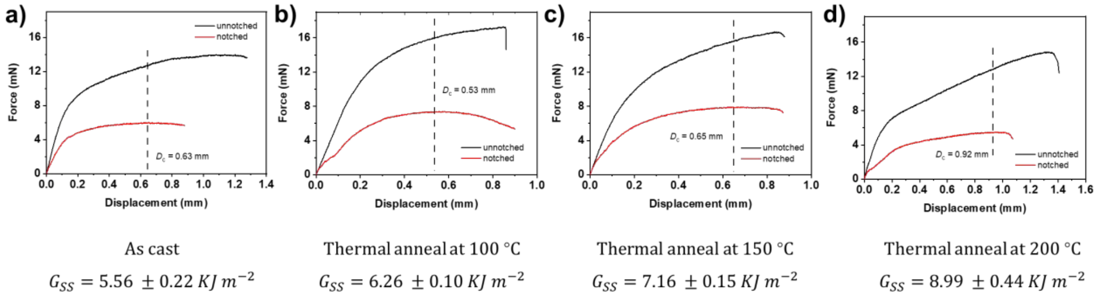

Figure S6. Force−displacement curves for unnotched and notched as cast and thermal annealed P(DPP-T-$C_2C_{12}C_{14}$) thin films at 100 °C, 150 °C and 200 °C. Molecular weight of the sample tested here is 75 kDa.

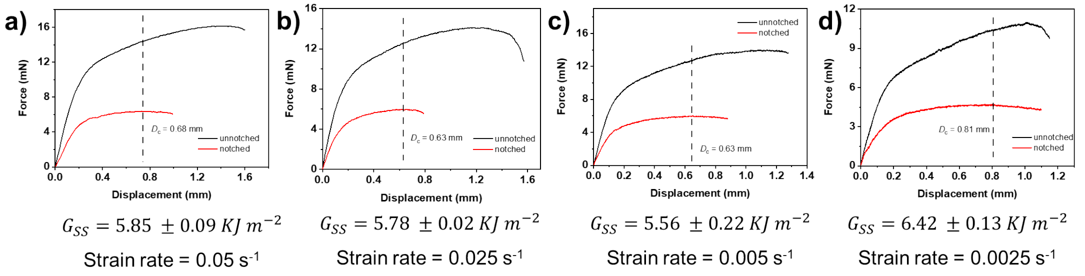

Figure S7. Force–displacement curves for unnotched and notched P(DPP-T-$C_2C_{12}C_{14}$) at strain rate of 0.0025, 0.005, 0.025 and 0.05 s^{-1}. Molecular weight of the sample tested here is 75 kDa.
